# Reliability and limits of transport-ventilators to safely ventilate severe patients in special surge situations

**DOI:** 10.1186/s13613-020-00782-5

**Published:** 2020-12-09

**Authors:** Dominique Savary, Arnaud Lesimple, François Beloncle, François Morin, François Templier, Alexandre Broc, Laurent Brochard, Jean-Christophe Richard, Alain Mercat

**Affiliations:** 1 Emergency Department, University Hospital of Angers, 4, Rue Larrey, 49933 Angers Cedex 9, France; 2 Inserm, EHESP, University of Rennes, Irset (Institut de Recherche en Santé, Environnement et Travail) - UMR_S 1085, 49000 Angers, France; 3 CNRS, INSERM 1083, MITOVASC, Université d’Angers, Angers, France; 4 Med2Lab, ALMS, Antony, France; 5 Critical Care Department, Angers University Hospital, Angers, France; 6 The Telecom-Physic-Strasbourg, Strasbourg University, Strasbourg , France; 7 Keenan Research Centre for Biomedical Science, Li Ka Shing Knowledge Institute, St. Michael’s Hospital, Toronto, Canada; 8 Interdepartmental Division of Critical Care Medicine, University of Toronto, Toronto, Canada; 9 INSERM, UMR 955 Eq 13, Toronto, Canada

**Keywords:** COVID-19, Acute Respiratory Distress Syndrome, Respiratory failure, Mechanical ventilation, Respiratory mechanics

## Abstract

**Background:**

Intensive Care Units (ICU) have sometimes been overwhelmed by the surge of COVID-19 patients. Extending ICU capacity can be limited by the lack of air and oxygen pressure sources available. Transport ventilators requiring only one O_2_ source may be used in such places.

**Objective:**

To evaluate the performances of four transport ventilators and an ICU ventilator in simulated severe respiratory conditions.

**Materials and methods:**

Two pneumatic transport ventilators, (Oxylog 3000, Draeger; Osiris 3, Air Liquide Medical Systems), two turbine transport ventilators (Elisee 350, ResMed; Monnal T60, Air Liquide Medical Systems) and an ICU ventilator (Engström Carestation—GE Healthcare) were evaluated on a Michigan test lung. We tested each ventilator with different set volumes (Vt_set_ = 350, 450, 550 ml) and compliances (20 or 50 ml/cmH_2_O) and a resistance of 15 cmH_2_O/l/s based on values described in COVID-19 Acute Respiratory Distress Syndrome. Volume error (percentage of Vt_set_) with P_0.1_ of 4 cmH_2_O and trigger delay during assist-control ventilation simulating spontaneous breathing activity with P_0.1_ of 4 cmH_2_O and 8 cmH_2_O were measured.

**Results:**

Grouping all conditions, the volume error was 2.9 ± 2.2% for Engström Carestation; 3.6 ± 3.9% for Osiris 3; 2.5 ± 2.1% for Oxylog 3000; 5.4 ± 2.7% for Monnal T60 and 8.8 ± 4.8% for Elisee 350. Grouping all conditions (P_0.1_ of 4 cmH_2_O and 8 cmH_2_O), trigger delay was 50 ± 11 ms, 71 ± 8 ms, 132 ± 22 ms, 60 ± 12 and 67 ± 6 ms for Engström Carestation, Osiris 3, Oxylog 3000, Monnal T60 and Elisee 350, respectively.

**Conclusions:**

In surge situations such as COVID-19 pandemic, transport ventilators may be used to accurately control delivered volumes in locations, where only oxygen pressure supply is available. Performances regarding triggering function are acceptable for three out of the four transport ventilators tested.

## Introduction

During the COVID 19 pandemic, several hospitals experienced the greatest shortage of ventilators ever seen since the heroic times of the polio epidemic in the 1950s. In this context, alternative solutions including ventilator sharing, use of anesthesia ventilators and use of homecare ventilators have been considered to manage intubated patients with severe lung failure outside the walls of the ICU [1–3]. To be able to replace ICU ventilators in the early phase, ventilators must be relatively easy for the users, able to accurately control the delivered volume and provide assist control ventilation (ACV) in difficult mechanical conditions. Importantly, they must allow to vary FiO_2_ without requiring two pressurized sources of gas (i.e., wall air and oxygen at 50 psi). Of note, this is one of the main limits of the homecare ventilators that makes them incompatible for very hypoxemic patients. Several transport ventilators are based on pneumatic systems and Venturi systems for gas mixing. Others use an internal turbine for pressurization; but need a pressurized gas source of oxygen to reach high FiO_2_ values. Pneumatic transport ventilators have been used for decades both for in- and out-of-hospital transport. Their robustness and their relative technological simplicity could potentially facilitate massive industrial production. They represent interesting solutions in this context and could fulfill the mentioned requirements. The general view on these ventilators is, however, that their limitations make them acceptable only for a short period like transport but make them incompatible with the safe delivery of difficult ventilation for very sick patients over prolonged periods. Undoubtedly, they have limited capacities regarding ventilation modes and monitoring, but knowing whether their reliability is sufficient for delivering lung protective ventilation in patients with ARDS merited to be tested with these objectives in mind. Indeed, discarding their use in a context of surge could limit the extension of beds outside the walls of the ICU for mechanically ventilated patients. Performances of turbine ventilators are often excellent and have been well described [4, 5]. By contrast, limits of pneumatic ventilators have not been specifically tested with the appropriate settings in realistic conditions simulating the respiratory mechanics of patients with COVID-19 induced ARDS [6–9].

The aim of the present study was to evaluate the reliability and the limitations of ventilation provided by these different technologies mimicking patients with COVID-19 induced ARDS in simulated bench conditions of passive and partially assisted situation.

## Materials and methods

Performances during volume-controlled (VC) and ACV were evaluated with different conditions of simulated respiratory mechanics reproducing patients with COVID-19 induced ARDS. All experiments have been performed in the Ventilatory Laboratory of the Angers University Hospital, medical ICU.

### Ventilators

#### Brands

Four transport ventilators necessitating only one O_2_ pressurized gas source were included in the study. Two pneumatic transport ventilators using Venturi systems to mix air to oxygen were tested: the Oxylog 3000 (Draeger, Lubeck, Germany) and the Osiris 3 (Air Liquide Medical Systems, Antony, France). Two turbine transport ventilators, necessitating additional oxygen only to increase FiO_2_ were also tested: the Elisee 350 (ResMed, Sydney, Australia) and the Monnal T60 (Air Liquide Medical Systems, Antony France). Performances of these ventilators were compared to a standard ICU ventilator: Engström Carestation (GE healthcare, Madison, USA). The characteristics of the five ventilators are given in Table 1.

**Table 1:** General characteristics of the ventilators

	Engström Carestation	Osiris 3	Oxylog 3000	Monnal T60	Elisee 350
Manufacturer	GE Healthcare	Air Liquide Medical Systems	Draeger	Air Liquide Medical Systems	Resmed
Weight [kg]	31.0	5.0	5.4	3.7	4.0
Working pressure	Pressurized oxygen and air	Pressurized oxygen	Pressurized oxygen	Pressurized oxygen	Pressurized oxygen
Expired volume monitoring	Yes	Yes	Yes	Yes	Yes
Tidal volume (Vt) [ml]	20–2000	100–2000	50–2000	20–2000	50–2500
Accuracy of Expiratory flow sensor	± 10%	± 15%	± 15%	VTe ≥ 50 ml: ± (2.5 ml ± 15%)	10% or 10 ml
PEEP [cmH_2_O]	1–50	0–15	0–20	0–20	0–25
Peak inspiratory pressure [cmH_2_O]	7–100	10–80	PEEP + 3–PEEP + 55	0–80	0–100
FiO_2_ [%]	21–100	70 or 100	40–100	21–100	21–100
Battery duration [h]	0.5–2	6–14	4	2.5–5	3–6

*PEEP* positive end expiratory pressure, *FiO*_*2*_ fraction of inspired oxygen

#### Working principle and settings

In the two pneumatic transport ventilators tested (Oxylog 3000 and Osiris 3), the working pressure that generates ventilation comes from the high-pressure oxygen supply. These ventilators based on a “Venturi-distributor” technology work as flow generator.

With the Oxylog 3000, the air-O_2_ mixing is regulated from 40 to 100% via a Venturi system coupled with a proportional inspiratory valve that also permits to directly set the volume (Vt_set_). The inspiratory flow depends on both the respiratory rate (RR) and the Inspiratory:Expiratory (I:E) ratio. In other words, for a given set volume, changing RR and/or I:E ratio keep the set Vt but modifies inspiratory flow. The monitoring of the expired Vt is available via a specific flow sensor inserted between the endotracheal tube and the patient circuit.

With the Osiris 3, only two positions are available for FiO_2_: 100% or 70%. A Venturi effect is used to obtain a FiO_2_ of 70% by mixing ambient air and O_2_ source. Inspiratory flow is delivered through a distributor. For a given combination of I:E ratio and respiratory rate, the Vt is set by directly adjusting a Vt knob that also regulates the inspiratory flow. The monitoring of the expired Vt is available via a specific flow sensor inserted between the endotracheal tube and the patient circuit.

The Elisee 350 and T60 are two turbine-based ventilators which need oxygen only to adjust FiO_2_. On those ventilators, the Vt and the inspiratory flow are directly set on the screen. Changing the respiratory rate does not affect neither Vt nor inspiratory flow. The monitoring of the expired Vt is available via a flow sensor close to the expiratory valve.

The Engström Carestation is a classical high-quality ICU ventilator requiring two sources of pressurized gas for oxygen and air (usually wall pressure at 50–55 psi). The monitoring of the expired Vt is available via a flow sensor located close to the expiratory valve.

### Volume delivered and PEEP with different respiratory mechanics

We assessed the volume effectively delivered (Vte_measured_) by the ventilators in different conditions of respiratory mechanics simulated on a Michigan test lung (Michigan Instruments, Kentwood, MI, USA). A linear pneumotachograph (PNT 3700 series, Shawnee, USA) and a pressure transducer (SD160 series: Biopac systems, Goleta, CA, USA) were used to measure flow and airway pressure between the test lung and the patient circuit. Signals were converted with an analog digital converter (MP150; Biopac systems, Goleta, CA, USA) at a sample rate of 200 Hz, and stored in a laptop using a dedicated software (Acknowledge, Biopac Systems). Vte_measured_ was obtained from numerical integration of the flow signal. All the tests were done in ATPD conditions and not corrected for BTPS conditions.

Three set volumes (Vt_set_) were tested: 350 ml, 450 ml and 550 ml, which approximately cover 6 ml/kg of Predicted Body Weight (PBW) for 161 to 199 cm height in male adult patients and 166 to 203 cm height in female adult patients. We also tested 300 mL on the Osiris 3. The different respiratory mechanics conditions tested were compliance of 50 ml/cmH_2_O and 20 ml/cmH_2_O, both combined with a resistance of 15 cmH_2_O/L/s. The combinations of compliance and resistance tested were based on recently described COVID-19 respiratory mechanics [6–9].

Assist Control Ventilation (ACV) mode was selected and similar ventilator settings were applied for each ventilator (respiratory rate 30 cycles/min).

The pneumatic transport ventilators were set with an Inspiratory:Expiratory ratio of 1:3 (I:E), whereas a flow of 60 l/min was adjusted on the Engström Carestation, Elisee 350 and Monnal T60. For every condition tested, inspiratory flow was measured.

The three set volumes were tested with FiO_2_ 100% and 70% as follows: FiO_2_ was selected, Vt_set_ was adjusted on the ventilator and Vte_measured_ was recorded and averaged over 5 cycles after stabilization. As Osiris 3 does not have an oxygen sensor to monitor oxygen content, FiO_2_ was measured on this ventilator when air-O_2_ mix was selected with a PF300 gas analyzer (IMT Medical, Buchs, Switzerland) in different conditions (Vt = 350–450–550 ml and Compliance = 20–50 ml/cmH_2_O).

The performances of Venturi-based ventilation in terms of volume delivery could be altered by set inspiratory flow values [10]. To assess in the Osiris 3 the impact of the inspiratory flow on Vte_error_, we tested a Vt_set_ of 450 ml obtained with different inspiratory flows achieved by changing respiratory rate (RR). ACV mode with air-O_2_ mix was selected, a resistance of 15 cmH_2_O/l/s and a compliance of 20 ml/cmH_2_O were applied and we set a I:E ratio of 1:3. The lowest RR (6 cycles/min) was chosen and was progressively increased by 4 cycles/min until reaching the maximum RR of 40 cycles/min. Vt_set_ had to be adjusted in consequence at each RR increment to keep its value at 450 ml. Vte_error_ was estimated at each step.

Two levels of PEEP were applied (10 cmH_2_O and 15 cmH_2_O) and the accuracy of the effective PEEP (PEEP_measured_) was assessed.

#### Volume error and PEEP end-points

The relative volume error (Vte_error_), which is the difference between the effective expired volume (Vte_measured_) and the set volume (Vt_set_) was calculated and averaged as previously described over the four different conditions [4, 11]:

$$ \begin{aligned}{\text{Resistance}}\, &= \, 1 5 {\text{ cmH}}_{ 2} {\text{O}}/{\text{l}}/{\text{s}},{\text{ Compliance}}\, \\ &= \, 20{\text{ or 5}}0 {\text{ml}}/{\text{cmH}}_{ 2} {\text{O}},{\text{ PEEP}}\, \\ &= \, 10{\text{ or 15 cmH}}_{ 2} {\text{O}}\end{aligned} $$

The relative volume error was expressed in percentage and defined as follows:

$$Vte_{error} = \frac{{\left| {Vte_{measured} - Vte_{set} } \right|}}{{Vte_{set} }} \times 100$$

*End-point for Vt*_*error*_ The three tidal volumes tested were chosen to cover theoretical “6 ml/kg PBW” in adult male or female patients (350, 450 and 550 ml correspond to 6 ml/kg PBW for, respectively 58, 75 and 92 kg PBW).

Ventilation was considered safe and acceptable when Vte_measured_ was within ± 0.5 ml/kg PBW, which covers a volume between 5.5 and 6.5 ml/kg PBW. This corresponds to an 8% difference between set and measured Vt.

*End-point for PEEP* A difference between measured PEEP and set PEEP was acceptable when less than 2 cmH_2_O.

#### Trigger performances

**Fig. 1:**
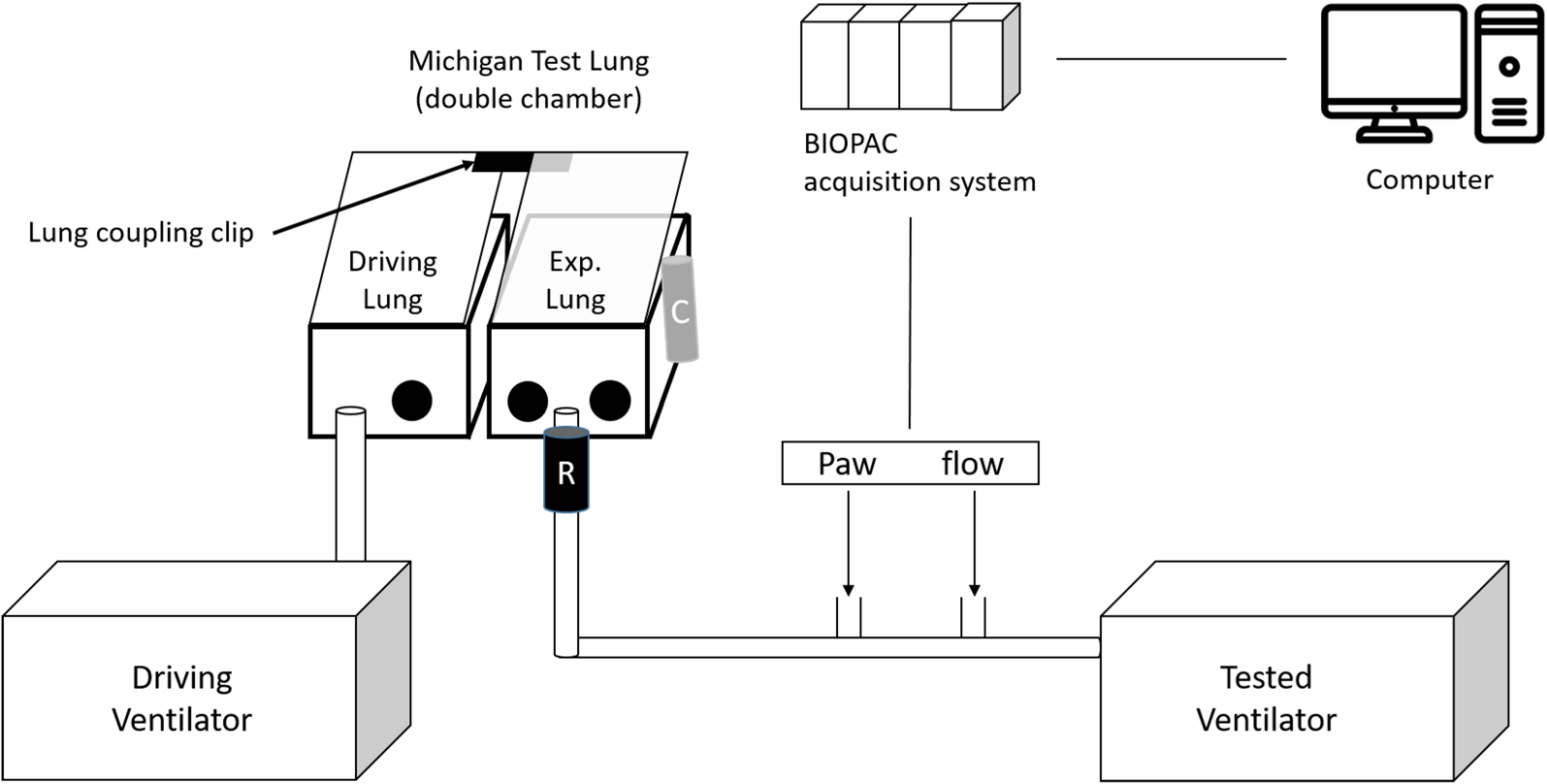
Illustration of bench test to simulate spontaneous breathing to assess trigger performances. The figure illustrates the bench test used to simulate spontaneous breathing to assess trigger performances. A double chamber Michigan test lung was used to simulate spontaneous breathing. One chamber of the test lung was defined as the driving lung while the other chamber was connected to the ventilator being tested. A lung-coupling clip allowed a connection between the two chambers, so that a positive pressure created in the driving lung (by the driving ventilator) induced a negative pressure in the experimental lung (“exp. Lung” on the figure), leading to trigger the ventilator tested. Of note, only one chamber of the test lung (experimental lung) is used to assess Vte error whereas the two chambers (driving lung and experimental lung) are used to simulate spontaneous breathing to assess trigger performances

Assist control ventilation (ACV) with the inspiratory trigger function “on” was tested by connecting ventilators to the double chamber Michigan test lung to simulate spontaneous breathing (see Fig. 1). One chamber of the test lung was defined as the driving lung, while the other chamber was connected to the ventilator being tested. A lung-coupling clip allowed a connection between the two chambers, so that a positive pressure created in the driving lung induced a negative pressure in the experimental lung, leading to trigger the ventilator tested.

The driving lung was connected to an Evita XL ventilator (Draeger, Lubeck, Germany), which was set in volume-controlled mode with constant flow. The respiratory rate was set at 25 breaths/min. The ventilatory settings were chosen to achieve a moderate effort, with a decrease in airway pressure 100 ms after occlusion (P_0.1_) of 4 cmH_2_O (consistent with P_0.1_ value recently described in COVID patients [12] measured at the airway opening of the lung model [13, 14]. A level of PEEP was applied to the driving lung to obtain a perfect contact of the lung-coupling clip between the two chambers at the end of expiration.

For each ventilator tested, volume assist-control ventilation (ACV) mode was selected, with a tidal volume of 450 ml, a respiratory rate of 20 cycles/min and a PEEP of 10 cmH_2_O. I:E ratio was set at 1:3 for Osiris 3 and Oxylog 3000, while a flow of 60 l/min was set on Elisee 350, Monnal T60 and the Engström Carestation ICU ventilator. Inspiratory triggers were set at their most responsive position while avoiding auto-triggering. The trigger of the Osiris 3 was set at − 0.5 cmH_2_O. Flow-triggered ventilators were set at 1 l/min for Engström Carestation and Oxylog 3000 and 2 l/min for Monnal T60 and Elisee 350. Two respiratory mechanics were tested: C = 20 ml/cmH_2_O and 50 ml/cmH_2_O with R = 15 cmH_2_O/l/s.

**Fig. 2:**
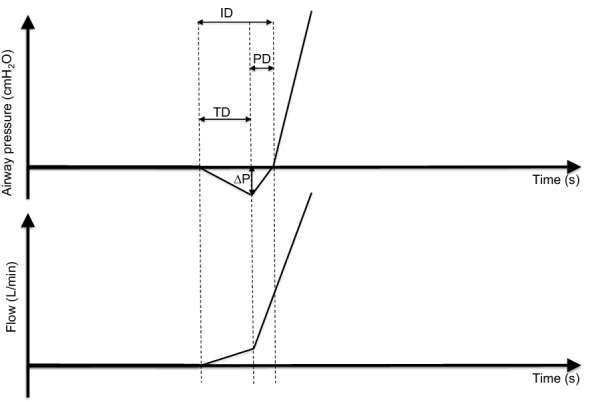
Explicative figure of ventilator triggering assessment. The figure illustrates ventilator triggering assessment. Airway pressure (Paw) and flow are displayed. Triggering delay (TD) is the delay between the onset of airway pressure drop (“patient” effort) and flow delivery by the ventilator. Pressurization delay (PD) is defined by the time at which the airway pressure comes back to the level of PEEP. The addition of TD and PD gives the inspiratory delay (ID). The drop of airway pressure (∆*P*) due to patient effort is also shown on the figure

For each configuration, trigger performance was assessed by measuring the airway pressure changes using the flow trace to determine the start of inspiration [11, 15]. Negative pressure drop (∆P, cmH_2_O), Triggering Delay (TD, ms) and Pressurization Delay (PD, ms) as defined in Fig. 2 were measured. The overall Inspiratory Delay (ID) corresponds to the addition of TD and PD.

We repeated the tests for all the ventilators at a strong effort corresponding to a P_0.1_ of 8 cmH_2_O; we tested the effect of set volume (Vt_set_ = 350–450–550 ml), compliance (C = 20–50 ml/cmH_2_O) and Positive End Expiratory Pressure (PEEP = 5–10–15 cmH_2_O) on triggering delay performances (see Additional file 1).

*End-point* Triggering function was considered as “safe and acceptable” when TD was less than 100 ms [16].

### Statistical analysis

Continuous variables were expressed as mean ± SD values averaged from 5 consecutive breaths. These variables were compared using an ANOVA test. The type I significance level was set at 0.05. When the global F was significant, post hoc tests were computed using a student *t* test with Bonferroni correction, which sets the level of significance for pairwise differences between the five ventilators at 0.005.

## Results

### Volume delivered and PEEP measured with different respiratory mechanics

**Fig. 3:**
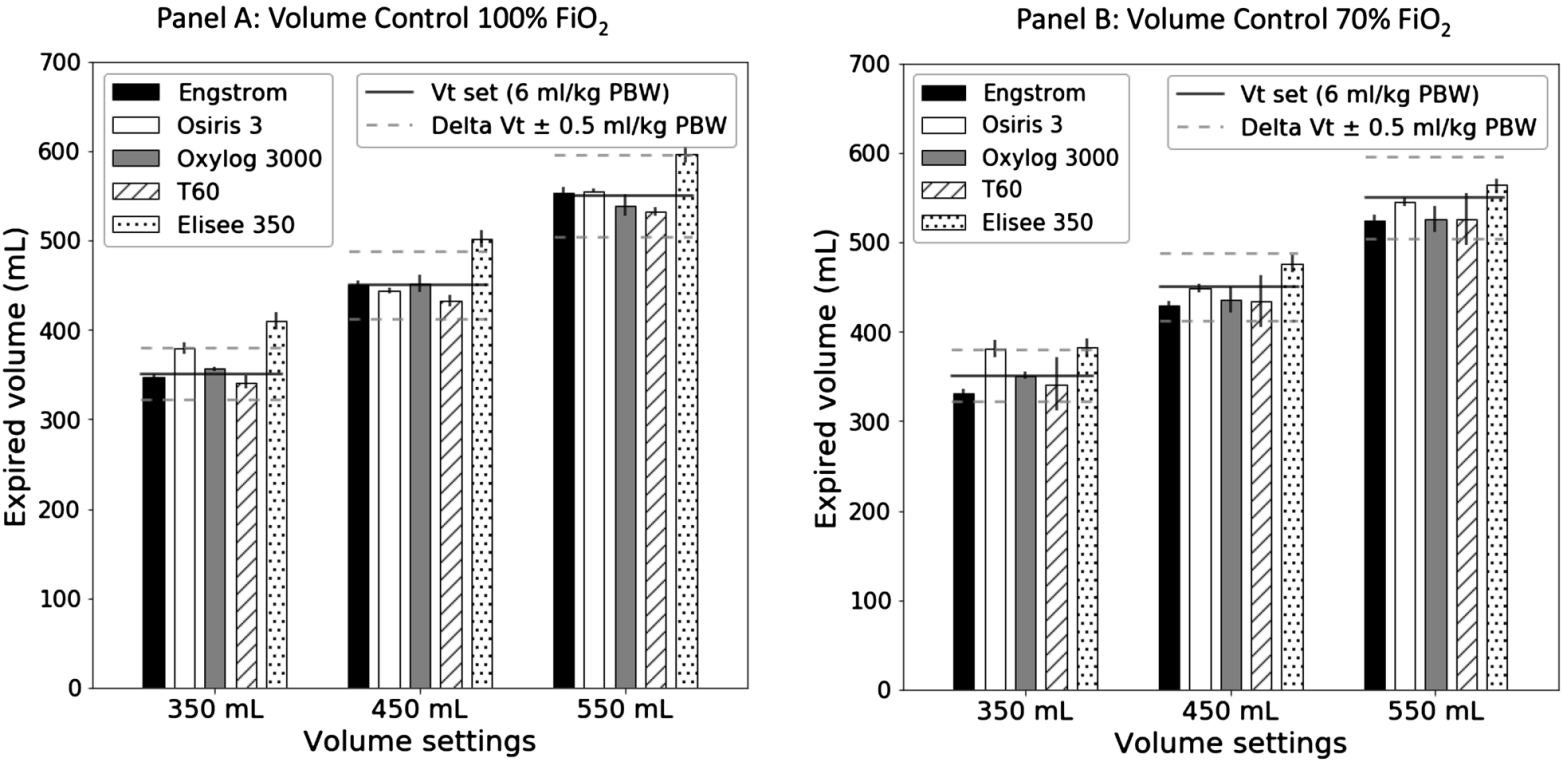
Tidal Volume delivery in volume control ventilation in static conditions. **a** The histogram represents the mean expired volumes measured for each ventilator according to the three Vt set in 100% FiO_2_. The average was computed over the four conditions of resistance (15 cmH_2_O/l/s), compliance (20–50 ml/cmH_2_O) and PEEP (10–15 cmH_2_O). The three tidal volumes tested were chosen to cover 6 ml/kg PBW, with 350, 450 and 550 ml corresponding to 6 ml/kg PBW for respectively 58, 75 and 92 kg PBW. Limits of acceptable ventilation are displayed with dotted lines and defined as a volume change within ± 0.5 ml/kg PBW, which corresponds to a Vt between 5.5 and 6.5 ml/kg PBW. **b** The histogram represents the mean expired volumes measured for each ventilator according to the three Vt set in 70% FiO_2_. The average was computed over the four conditions of resistance (15 cmH_2_O/l/s), compliance (20–50 ml/cmH_2_O) and PEEP (10–15 cmH_2_O)

Results obtained with a P0.1 of 4 cmH_2_O (moderate effort) are displayed in Fig. 3 and mean volume errors (Vte_error_) for each ventilator are shown in Table 2. When all conditions and set volumes were included, the Engström Carestation was the most accurate ventilator, and the Oxylog 3000 was comparable. The performance was considered as acceptable (delta Vt ± 0.5 ml/kg PBW) except for one turbine ventilator (Elisee 350). The impact of FiO_2_ selection (FiO2 100% or 70%) on volume error was significant considering all ventilators (*p* < 0.05, see Table 2). There was no impact of compliance on volume error (*p* > 0.05, Table 2). FiO_2_ measured on Osiris 3 was 72.3 ± 1.7% across all the conditions tested. Differences between measured PEEP and set PEEP were less than 2 cmH_2_O as shown in Table 2 (all conditions together).

**Table 2:** Mean volume errors and Positive End Expiratory Pressure 1 (PEEP) measured for each ventilator

	Engström Carestation	Osiris 3	Oxylog 3000	Monnal T60	Elisee 350
Vte_error_ global [%]	2.9 ± 2.2	3.6 ± 3.9	2.5 ± 2.1	5.4 ± 2.7 (*)	8.8 ± 4.8 (*)
Vte_error_ 100% FiO_2_ [%]	1.0 ± 0.7	3.7 ± 3.7 (*)	2.0 ± 1.2 (*)	3.3 ± 1.4 (*)	11.9 ± 4.1 (*)
Vte_error_ 70% FiO_2_ [%]	4.9 ± 1.3	3.5 ± 4.2	2.9 ± 2.7 (*)	7.5 ± 2.0 (*)	5.9 ± 3.5
Vte_error_ C50 [%]	3.3 ± 2.7	3.4 ± 4.0	1.6 ± 1.1 (*)	5.1 ± 3.1 (*)	10.4 ± 5.3 (*)
Vte_error_ C20 [%]	2.6 ± 1.6	3.8 ± 3.8	3.4 ± 2.6	5.7 ± 2.2 (*)	7.0 ± 3.7 (*)
Mean PEEP 10 [cmH_2_O]	9.9 ± 0.2	10.6 ± 0.6 (*)	11.5 ± 0.3 (*)	9.5 ± 0.5 (*)	10.3 ± 0.1 (*)
Mean PEEP 15 [cmH_2_O]	15.1 ± 0.2	14.9 ± 0.6 (*)	15.4 ± 2.2	14.7 ± 0.1 (*)	15.4 ± 0.2 (*)

*Vte*_*error*_ *global *= mean volume error including all conditions of resistance (15 cmH_2_O/l/s), compliance (20–50 ml/cmH_2_O) and PEEP (10–15 cmH_2_O) for both 100% FiO_2_ and 70% FiO_2_. *Vte*_*error*_ *100% FiO*_*2*_= mean volume error including all conditions of resistance (15 cmH_2_O/l/s), compliance (20–50 ml/cmH_2_O) and PEEP (10–15 cmH_2_O) for 100% FiO_2_, *Vteerror 70% FiO*_*2*_= mean volume error including all conditions of resistance (15–12 cmH_2_O/l/s), compliance (20–50 ml/cmH_2_O) and PEEP (10–15 cmH_2_O) for 70% FiO_2_. *Vte*_*error*_ *C50 *= mean volume error including all conditions of 1 resistance (15 cmH_2_O/l/s), FiO_2_ (100–70%) and PEEP (10–15 cmH_2_O) for a compliance of 50 ml/cmH_2_O. *teerror C20 *= mean volume error including all conditions of resistance (15 cmH_2_O/l/s), FiO_2_ (100–70%) and PEEP (10–15 cmH_2_O) for a compliance of 20 ml/cmH_2_O, *Mean PEEP 10 *= mean PEEP measured when PEEP was set at 10 cmH_2_O including all conditions of resistance (15 cmH_2_O/l/s) and compliance (20–50 ml/cmH_2_O) for both 100% FiO_2_ and 70% FiO_2_. *Mean PEEP 15 *= mean PEEP measured when PEEP was set at 15 cmH_2_O including all conditions of resistance (15 cmH_2_O/l/s) and compliance (20–50 ml/cmH2O) for both 100% FiO_2_ and 70% FiO_2_

*p < 0.005 when comparing each transport ventilator with the Engstrom ICU ventilator (ANOVA test: global F was significant)

Volume delivered at a Vt_set_ of 300 ml for the Osiris 3 is shown in Additional file 1: Figure S1. Additional file 1: Table S1 summarizes measured inspiratory flow for each ventilator in the different experimental conditions.

#### Impact of inspiratory flow on pneumatic ventilators

**Fig. 4:**
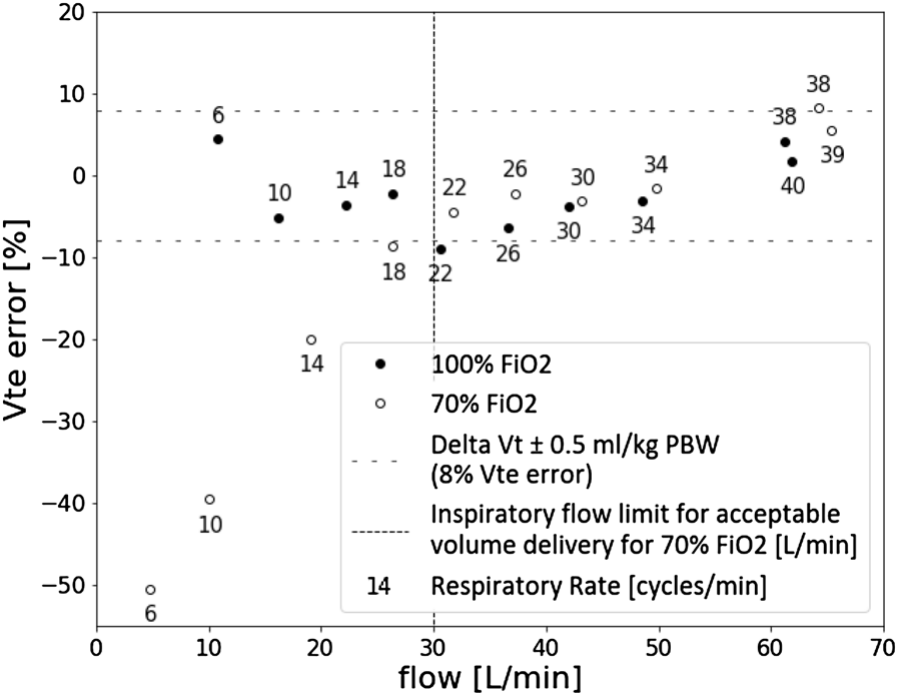
Impact of flow on effective volume with Osiris 3 ventilator. This figure shows the volume error of the Osiris 3 expressed in percentage of Vt set according to different inspiratory flows obtained at a constant 450 ml Vt set. Compliance, resistance and PEEP were set at 20 ml/cmH_2_O, 15 cmH_2_O/l/s and 10 cmH_2_O respectively. Black circles were obtained with 100% FiO_2_ while the white circles were obtained with 70% FiO_2_. Respiratory rate associated with each point is also displayed. This figure illustrates that for an inspiratory flow below 30 l/min, the Vt error is substantial with 70% FiO_2_. The Vt error is within ± 0.5 ml/kg PBW (which corresponds to an 8% difference between set and measured Vt) whatever the inspiratory flow when 100% FiO_2_ is selected

The effect of inspiratory flow rates on Vte_error_ for Osiris 3 is shown in Fig. 4. Considering a Vt_set_ of 450 ml, the lowest values of inspiratory flow were associated with a Vte_error_ higher than 8% (delta Vt ± 0.5 ml/kg PBW). Performances were acceptable when inspiratory flow (resulting from the combination of Vt, I:E ratio and respiratory rate) was strictly above 30 l/min, which corresponds to a respiratory rate higher than 18 cycles/min.

#### Trigger performances during ACV

**Fig. 5:**
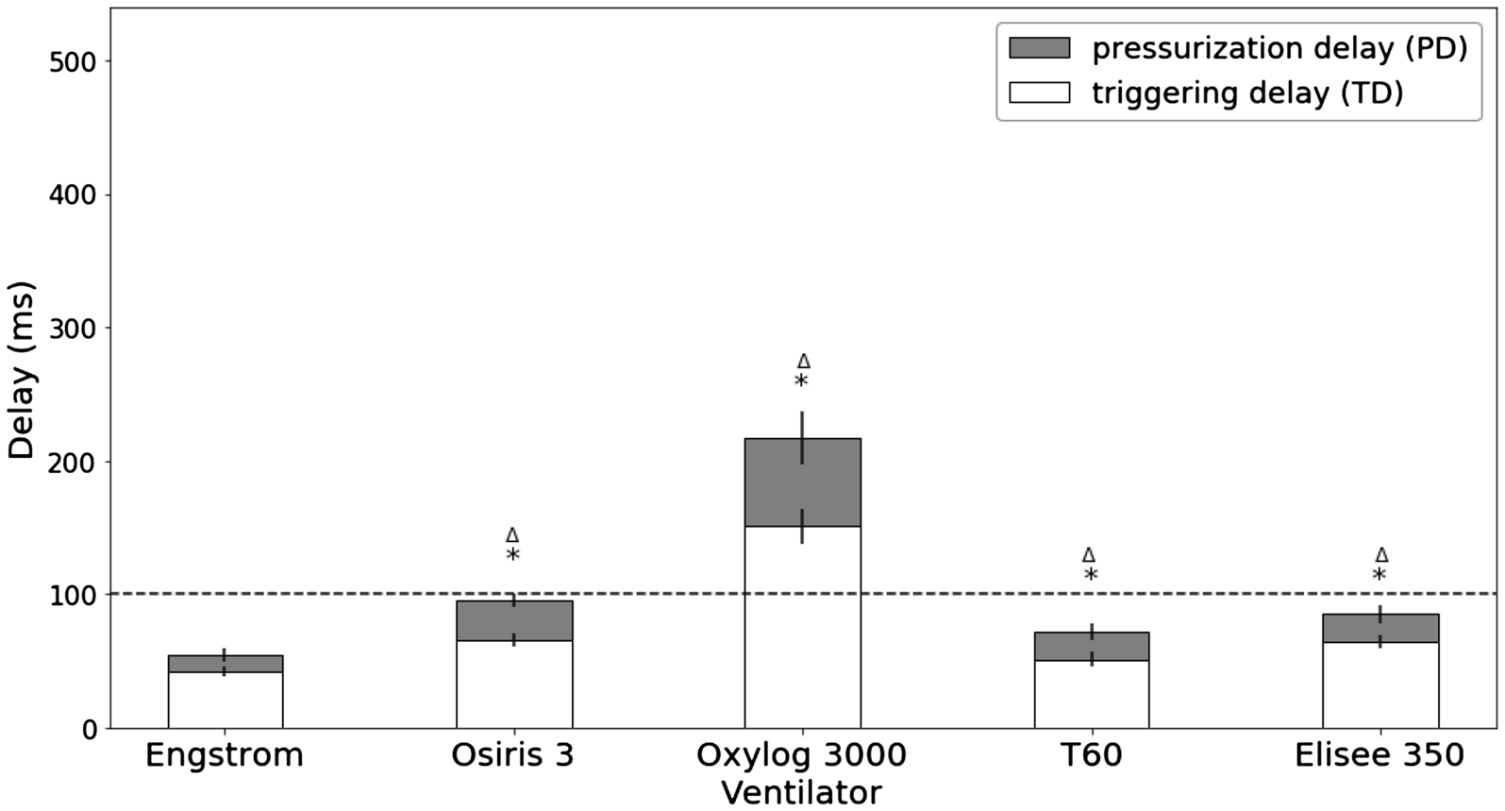
Triggering characteristics in volume assist-control ventilation for a P0.1 of 4 cmH_2_O. The figure illustrates the triggering efficiency for each ventilator tested during assist-control ventilation using the Michigan test lung to simulate spontaneous breathing. A moderate effort was achieved, corresponding to a decrease in airway pressure 100 ms after occlusion (P0.1) of 4 cmH_2_O was achieved. A PEEP of 10 cmH_2_O, a compliance of 20 and 50 ml/cmH_2_O and a resistance of 15 cmH_2_O/l/s were selected. Triggering Delay (TD, ms) and Pressurization Delay (PD, ms) were computed. A definition of TD and PD is available on Fig. 1. Triggering function was considered safe and acceptable when TD was less than 100 ms. *p < 0.005 for TD when comparing each transport ventilator with the Engstrom ICU ventilator (ANOVA test: global F was significant). ^Δ^*p* < 0.005 for PD when comparing each transport ventilator with the Engstrom ICU ventilator (ANOVA test: global F was significant)

Inspiratory trigger was evaluated for each ventilator and results corresponding to a moderate effort (P_0.1_ = 4 cmH_2_O) are displayed in Fig. 5. All simulated efforts triggered a ventilatory cycle. The Triggering Delay was 42 ± 4 ms, 65 ± 5 ms, 151 ± 14 ms, 51 ± 6 ms and 64 ± 5 ms for Engström Carestation, Osiris 3, Oxylog 3000, Monnal T60 and Elisee 350, respectively (all conditions grouped, *p* < 0.05; pairwise differences between ventilators were all significant with a *p*-value < 0.005). The Inspiratory Delay (ID) was measured at 54 ± 5 ms for Engström Carestation, 95 ± 5 ms for Osiris 3, 217 ± 21 ms for Oxylog 3000, 72 ± 6 ms for Monnal T60 and 85 ± 7 ms for Elisee 350 and (*p* < 0.05; pairwise differences between ventilators were all significant with a *p*-value < 0.005).

The airway pressure drop was much larger for Oxylog 3000 (− 4.2 ± 0.3 cmH_2_O), than for the others: − 0.9 ± 0.3 cmH_2_O for Engström Carestation, − 1.9 ± 0.1 cmH_2_O for Osiris 3, − 0.6 ± 0.1 cmH_2_O for Monnal T60 and − 0.8 ± 0.1 for Elisee 350 (*p* < 0.05; pairwise differences between ventilators were all significant with a *p*-value < 0.005, except between Engstrom Carestation-Elisee 350 and Engstrom Carestation-Monnal T60).

Ventilator performances were considered acceptable (TD < 100 ms) except for one pneumatic ventilator (Oxylog 3000).

Triggering Delays obtained with a strong effort (P_0.1_ = 8 cmH_2_O) are available in Additional file 1: Figure S2. Grouping all conditions (P_0.1_ of 4 cmH_2_O and 8 cmH_2_O), trigger delay was 50 ± 11 ms, 71 ± 8 ms, 132 ± 22 ms, 60 ± 12 and 67 ± 6 ms for Engström Carestation, Osiris 3, Oxylog 3000, Monnal T60 and Elisee 350, respectively. The effect of set volume, compliance and PEEP on triggering delay performances (at P_0.1_ = 8 cmH_2_O) are shown in Additional file 1: Tables S2, S3 and S4, respectively.

## Discussion

The results of the present bench test study comparing turbine and pneumatic transport ventilators to an ICU ventilator, can be summarized as follows: 1. Turbine ventilators’ performances in VC and ACV are very close to those of the ICU ventilator tested for most of the settings including volume delivery and reliability of PEEP. 2. For most of the severe respiratory mechanics conditions tested, the volume error does not exceed 0.5 ml/kg PBW except for one turbine ventilator (two conditions) and one pneumatic ventilator (one condition). Volume error delivered by the simplest pneumatic ventilator significantly increased at FiO_2_ 70%, when inspiratory flow was less than 30 l/min indicating a technological limit of the Venturi system. 3. Inspiratory trigger reactivity was less than 100 ms except for one pneumatic transport ventilator.

The increasing number of patients requiring mechanical ventilation in the context of the COVID-19 worldwide crisis, and the ventilators shortage reported in some severely affected countries, has led to discuss the possibilities to manage intubated patients outside the walls of the ICU [2]. According to this dire scenario, simple and easy to set ventilators that only require one oxygen pressure source to function and able to reliably deliver lung protective ventilation could be considered. In addition, an assisted mode that controls the Vt with PEEP up to 15 cmH_2_O and FiO_2_ up to 100% is required to manage patients with high elastic load and severe shunt that characterize potentially severe COVID-19 ARDS [1, 2].

### Performances during controlled ventilation

Recent turbine transport and emergency ventilators display performances which are very close to conventional ICU ventilators [5, 17]. In the context of “mass casualty”, as experienced with the COVID-19 crisis, pneumatic transport ventilators could be used to extend the possibility to manage intubated patients in case of ICU beds shortage. The working principle of these pneumatic ventilators is based on a “Venturi system” which is a simple technological solution that permits to manage ventilation generated by the oxygen pressurized source when a position called air-O_2_ mix is selected. Interestingly, the simplicity of such pneumatic systems permits to consider massive industrialization faster and at a lower cost. On the opposite, the Venturi system explains the limits observed with low inspiratory flow previously described with this technology [10].

For pneumatic ventilators, in case of high impedance, a low inspiratory flow may increase significantly volume error when the air-O_2_ mix position is selected “on”. In turn, manipulating I:E ratio, respiratory rate and increasing inspiratory flow above 30 l/min permits to reverse the Vt error that is directly explained by the working principle of this ventilator (see Fig. 4). The technological adaptations available on Oxylog 3000 (Venturi coupled with proportional inspiratory valve) solve this problem, while expired Vt monitoring available on Osiris 3 simplifies settings adaptation if required. Previous bench test studies have reported a Vt error with pneumatic basic transport ventilators that reached 20% of set Vt with resistive load [10, 18]. These experiments were performed with very low set inspiratory flow thus explaining the Vt reduction observed. For clinical practice, when FiO_2_ 70% is used on the Osiris 3, an essential recommendation is to follow these steps: first adjust the I:E ratio at 1:3 (i.e., the minimal available value) and the respiratory rate at 18/min or above. Secondarily, the Vt knob that also controls the inspiratory flow must be adjusted to reach the desired Vt based on Vt expired monitoring. With these recommendations, volume error measured on pneumatic transport ventilators at low compliance is close from turbine performances and acceptable.

Of note, only the ICU and turbine ventilators tested compensate for the loss in Vt due to the compression of gas inside the circuit. Nevertheless, this effect previously quantified in ICU ventilators with inspiratory–expiratory circuits is significantly less in basic transport ventilators, since they are equipped with a single limb circuit [4]. Of note, an HEPA filter can be easily adjusted on the expiratory limb to limit risks of viral contamination.

### Performances during assisted ventilation

Recent experience with COVID-19 induced ARDS reports that these patients often exhibit high respiratory drive and asynchrony that may require deep sedation and sometimes paralysis [7]. We, therefore, evaluated the behavior of the four transport ventilators during triggered breaths, especially pneumatic ones, since performances of their trigger have been questioned [5, 18]. The triggering performances were acceptable except for the Oxylog 3000 exhibiting the poorest triggering performances. The triggering delay was consistently longer in pneumatic ventilators but acceptable except on the Oxylog 3000, compared to the ICU ventilator [5].

## Limitations

The results obtained in vitro necessitate some caution to be translated to the clinical practice, but previous studies showed that this type of simulation predicts the results observed in clinical situations with a high fidelity [4, 19, 20]. The lung model gives the unique opportunity to compare ventilator performances according to several simulated but standardized clinical conditions. Bench experiment also permits to accurately depict and understand advantages and limits of the different ventilator’s technologies as previously done [10]. The Michigan test lung (Michigan Instruments, Kentwood, MI, USA) used in the present study is a simple model that presents obvious limitations, but its reliability for Vt and trigger performances evaluation has been well demonstrated. Of note, the results have not been corrected in BTPS conditions, which may have slightly underestimated actual expired volumes [17]. Our experiment reported performances of only two pneumatic and two turbine ventilators, while several other ventilators with similar technology are available worldwide. We did not evaluate pressure support ventilation, while this approach can be useful to manage weaning of COVID-19 patients. Previous studies already showed that turbine-based ventilator significantly outperform pneumatic transport ventilators during pressure mode ventilation [5, 17].

Performances of pneumatic ventilators can be viewed as “acceptable” during the initial phase of respiratory failure. For patients with difficulties to be separated from the ventilator, better performances may be expected for assisted ventilation.

## Conclusion

The present bench study suggests that turbine technologies may acceptably replace ICU ventilators, at least transiently, to extend ICU beds, where only oxygen pressure supply is available, in special surge situations such as COVID-19 crisis. Pneumatic transport ventilators are limited in terms of FiO_2_ settings, but provide acceptable volume accuracy in severe simulated conditions. For this purpose, the respiratory rate should be set at or above 18/min (to maintain sufficient inspiratory flow) in the Osiris 3 with a FiO_2_ of 70% [21]. A monitoring of expired Vt available on the two pneumatic transport ventilators tested greatly facilitates adequate settings. Performances regarding triggering function are non-acceptable in one of the pneumatic transport ventilators.

## Supplementary information

**Additional file 1.** Addiional tables and figures.

## Data Availability

The datasets analyzed during the current study are available from the corresponding author on reasonable request.

## Abbreviations

ACV: Assist-Control Ventilation
ARDS: Acute Respiratory Distress Syndrome
COVID-19: Coronavirus Disease 2019
C_RS_: Compliance of the respiratory system
FiO_2_: Fraction of inspired oxygen
ICU: Intensive Care Unit
PEEP: Set Positive End-Expiratory Pressure
SARS-CoV-2: Severe Acute Respiratory Syndrome Coronavirus-2
VC: Volume control
Vt: Tidal volume

## References

[1] Beitler JR, Mittel AM, Kallet R, Kacmarek R, Hess D, Branson R (2020). Ventilator sharing during an acute shortage caused by the COVID-19 pandemic. Am J Respir Crit Care Med..

[2] Emanuel EJ, Persad G, Upshur R, Thome B, Parker M, Glickman A (2020). Fair allocation of scarce medical resources in the time of Covid-19. N Engl J Med.

[3] Monti G, Cremona G, Zangrillo A, Lombardi G, Sartini C, Sartorelli M (2020). Home ventilators for invasive ventilation of patients with COVID-19. Crit Care Resuscitation..

[4] Lyazidi A, Thille AW, Carteaux G, Galia F, Brochard L, Richard JCM (2010). Bench test evaluation of volume delivered by modern ICU ventilators during volume-controlled ventilation. Intensive Care Med..

[5] L’Her E, Roy A, Marjanovic N (2014). Bench-test comparison of 26 emergency and transport ventilators. Crit Care..

[6] Pan C, Chen L, Lu C, Zhang W, Xia J-A, Sklar MC (2020). Lung Recruitability in SARS-CoV-2 associated acute respiratory distress syndrome: a single-center, observational study. Am J Respir Crit Care Med..

[7] Gattinoni L, Coppola S, Cressoni M, Busana M, Rossi S, Chiumello D (2020). Covid-19 Does Not Lead to a “Typical” Acute Respiratory Distress Syndrome. Am J Respir Crit Care Med..

[8] Gattinoni L, Chiumello D, Caironi P, Busana M, Romitti F, Brazzi L (2020). COVID-19 pneumonia: different respiratory treatments for different phenotypes?. Intensive Care Med..

[9] Grasselli G, Zangrillo A, Zanella A, Antonelli M, Cabrini L, Castelli A (2020). Baseline characteristics and outcomes of 1591 patients infected with SARS-CoV-2 Admitted to ICUs of the Lombardy Region, Italy. JAMA.

[10] Breton L, Minaret G, Aboab J, Richard J-C (2002). Fractional inspired oxygen on transport ventilators: an important determinant of volume delivery during assist control ventilation with high resistive load. Intensive Care Med..

[11] Boussen S, Gainnier M, Michelet P (2013). Evaluation of ventilators used during transport of critically ill patients: a bench study. Respir Care..

[12] Gattinoni L, Marini JJ, Camporota L (2020). The respiratory drive: an overlooked tile of COVID-19 pathophysiology. Am J Respir Crit Care Med.

[13] Beloncle F, Piquilloud L, Olivier P-Y, Vuillermoz A, Yvin E, Mercat A (2019). Accuracy of P01 measurements performed by ICU ventilators: a bench study. Ann Intensive Care..

[14] Telias I, Junhasavasdikul D, Rittayamai N, Piquilloud L, Chen L, Ferguson ND (2020). Airway occlusion pressure as an estimate of respiratory drive and inspiratory effort during assisted ventilation. Am J Respir Crit Care Med.

[15] Richard J-C, Carlucci A, Breton L, Langlais N, Jaber S, Maggiore S (2002). Bench testing of pressure support ventilation with three different generations of ventilators. Intensive Care Med.

[16] Aslanian P, ElAtrous S, Isabey D, Valente E, Corsi D, Harf A (1998). Effects of flow triggering on breathing effort during partial ventilatory support. Am J Respir Crit Care Med..

[17] Thille AW, Lyazidi A, Richard JCM, Galia F, Brochard L (2009). A bench study of intensive-care-unit ventilators: new versus old and turbine-based versus compressed gas-based ventilators. Intensive Care Med..

[18] Zanetta G, Robert D, Guérin C (2002). Evaluation of ventilators used during transport of ICU patients—a bench study. Intensive Care Med.

[19] Carteaux G, Lyazidi A, Cordoba-Izquierdo A, Vignaux L, Jolliet P, Thille AW (2012). Patient-ventilator asynchrony during noninvasive ventilation. Chest..

[20] Vignaux L, Tassaux D, Jolliet P (2007). Performance of noninvasive ventilation modes on ICU ventilators during pressure support: a bench model study. Intensive Care Med..

[21] Garnier M, Quesnel C, Fulgencio J-P, Degrain M, Carteaux G, Bonnet F (2015). Multifaceted bench comparative evaluation of latest intensive care unit ventilators. Br J Anaesth.

