# Supplementary material for: Reliability and limits of transport-ventilators to safely ventilate severe patients in special surge situations
Source: Ann Intensive Care. 2020 Dec 9;10:166. doi: 10.1186/s13613-020-00782-5 (PMC7724620; doi:10.1186/s13613-020-00782-5)
Supplement: Supplementary file 1 — Additional file 1. Addiional tables and figures. [file 13613_2020_782_MOESM1_ESM.pptx]

## Slide 1
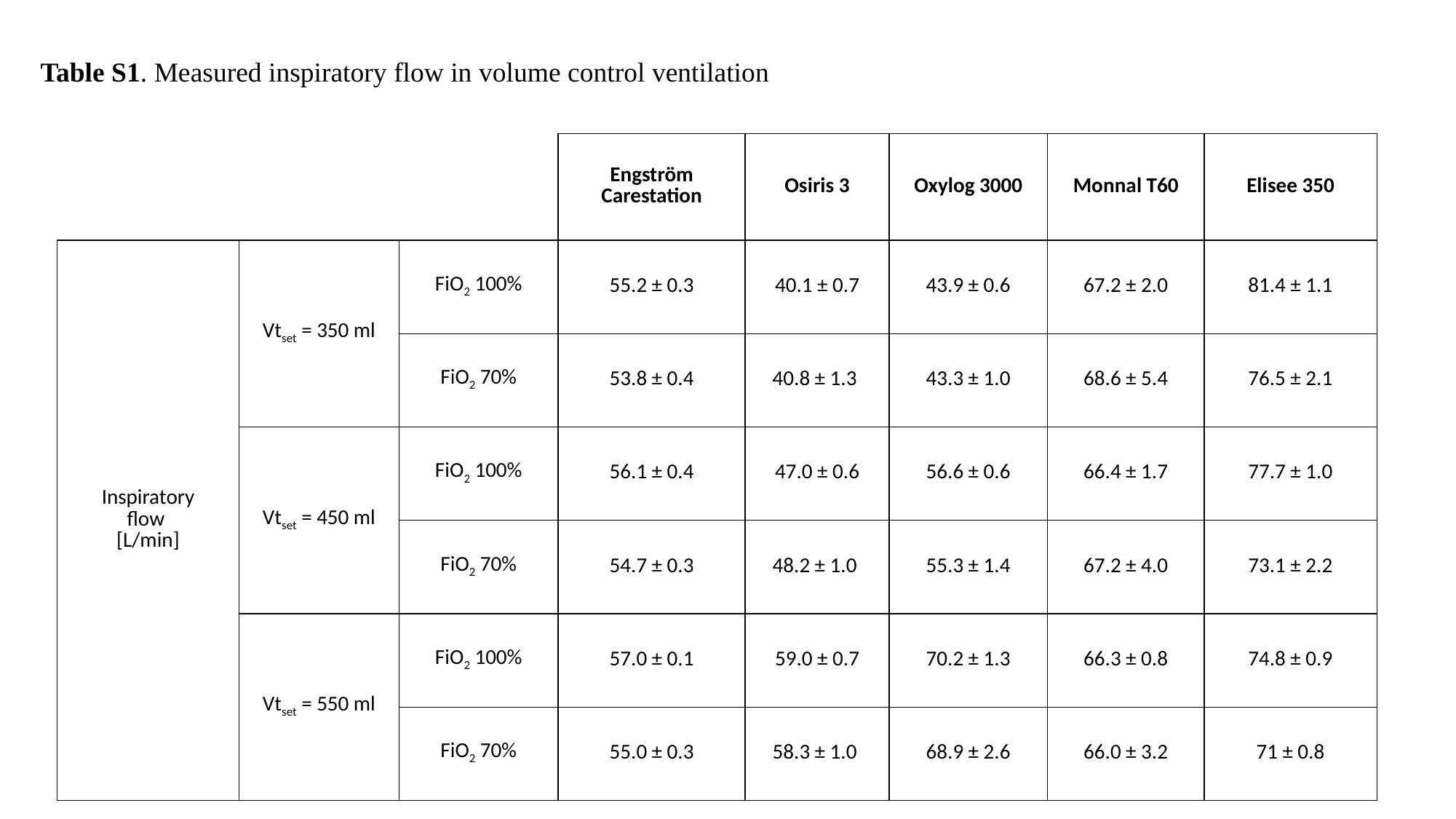

Table S1. Measured inspiratory flow in volume control ventilation
| | | | Engström Carestation | Osiris 3 | Oxylog 3000 | Monnal T60 | Elisee 350 |
| --- | --- | --- | --- | --- | --- | --- | --- |
| Inspiratory flow [L/min] | Vtset = 350 ml | FiO2 100% | 55.2 ± 0.3 | 40.1 ± 0.7 | 43.9 ± 0.6 | 67.2 ± 2.0 | 81.4 ± 1.1 |
| | | FiO2 70% | 53.8 ± 0.4 | 40.8 ± 1.3 | 43.3 ± 1.0 | 68.6 ± 5.4 | 76.5 ± 2.1 |
| | Vtset = 450 ml | FiO2 100% | 56.1 ± 0.4 | 47.0 ± 0.6 | 56.6 ± 0.6 | 66.4 ± 1.7 | 77.7 ± 1.0 |
| | | FiO2 70% | 54.7 ± 0.3 | 48.2 ± 1.0 | 55.3 ± 1.4 | 67.2 ± 4.0 | 73.1 ± 2.2 |
| | Vtset = 550 ml | FiO2 100% | 57.0 ± 0.1 | 59.0 ± 0.7 | 70.2 ± 1.3 | 66.3 ± 0.8 | 74.8 ± 0.9 |
| | | FiO2 70% | 55.0 ± 0.3 | 58.3 ± 1.0 | 68.9 ± 2.6 | 66.0 ± 3.2 | 71 ± 0.8 |

## Slide 2
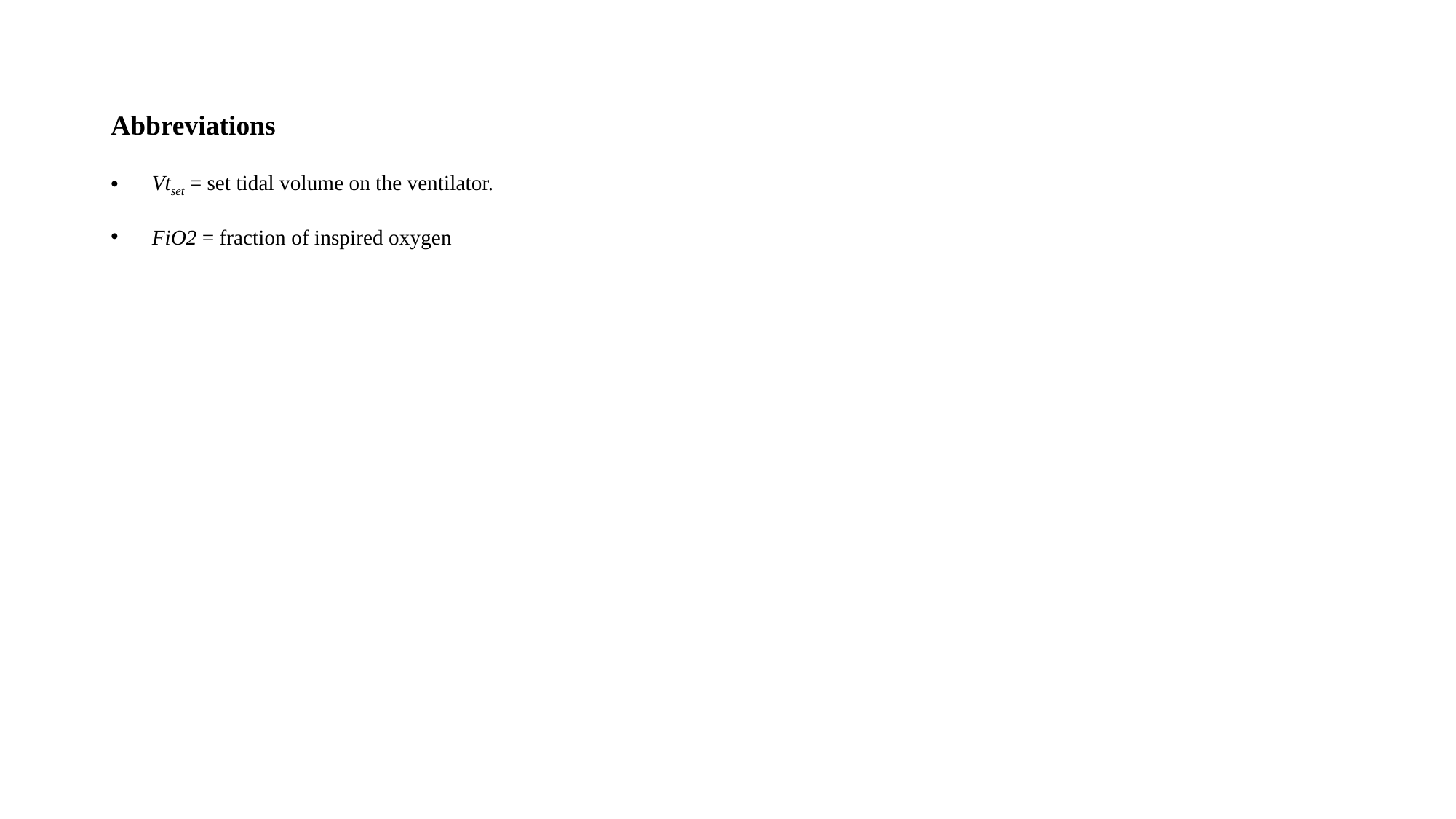

Abbreviations
Vtset = set tidal volume on the ventilator.
FiO2 = fraction of inspired oxygen

## Slide 3
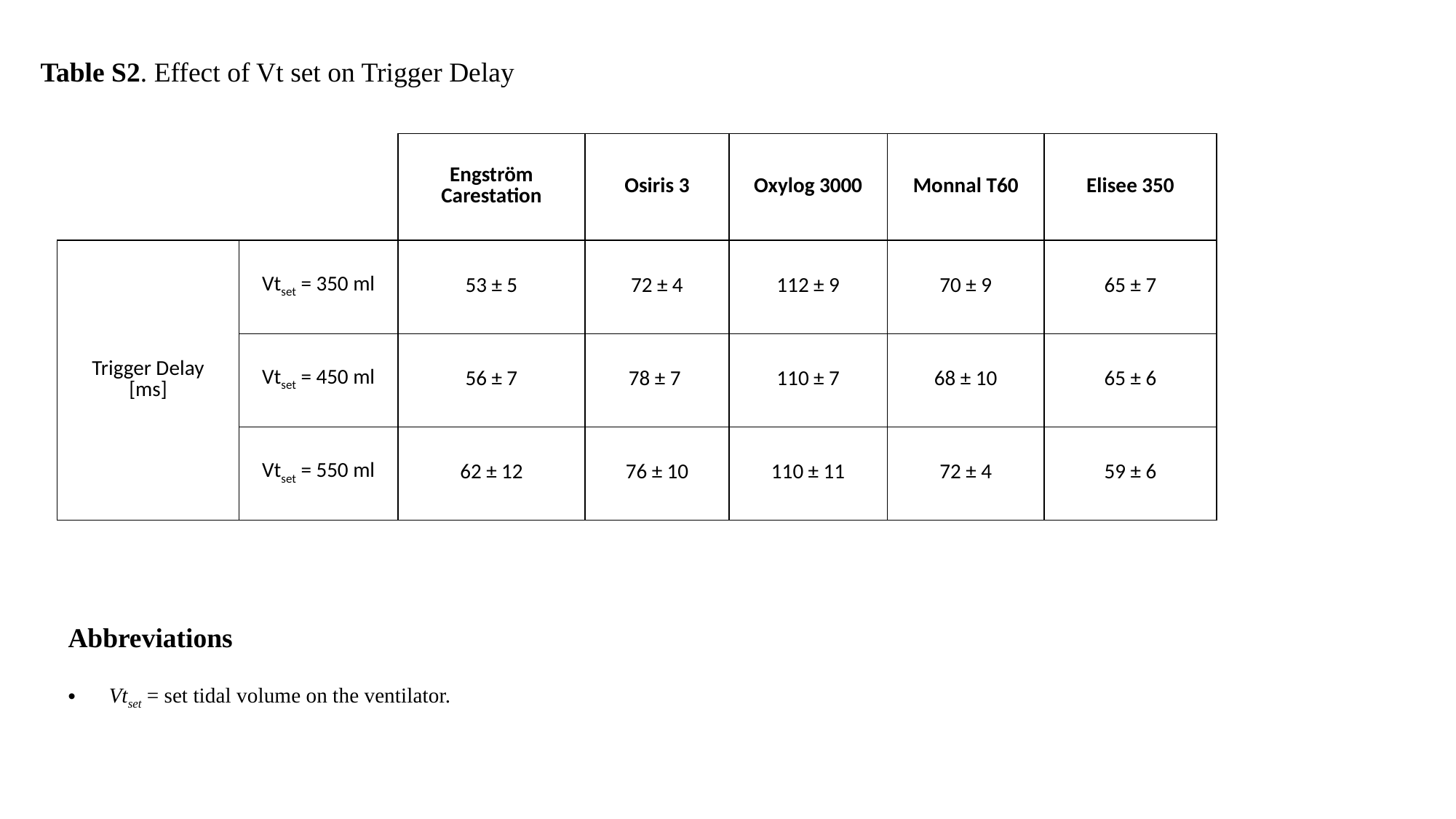

Table S2. Effect of Vt set on Trigger Delay
| | | Engström Carestation | Osiris 3 | Oxylog 3000 | Monnal T60 | Elisee 350 |
| --- | --- | --- | --- | --- | --- | --- |
| Trigger Delay [ms] | Vtset = 350 ml | 53 ± 5 | 72 ± 4 | 112 ± 9 | 70 ± 9 | 65 ± 7 |
| | Vtset = 450 ml | 56 ± 7 | 78 ± 7 | 110 ± 7 | 68 ± 10 | 65 ± 6 |
| | Vtset = 550 ml | 62 ± 12 | 76 ± 10 | 110 ± 11 | 72 ± 4 | 59 ± 6 |
Abbreviations
Vtset = set tidal volume on the ventilator.

## Slide 4
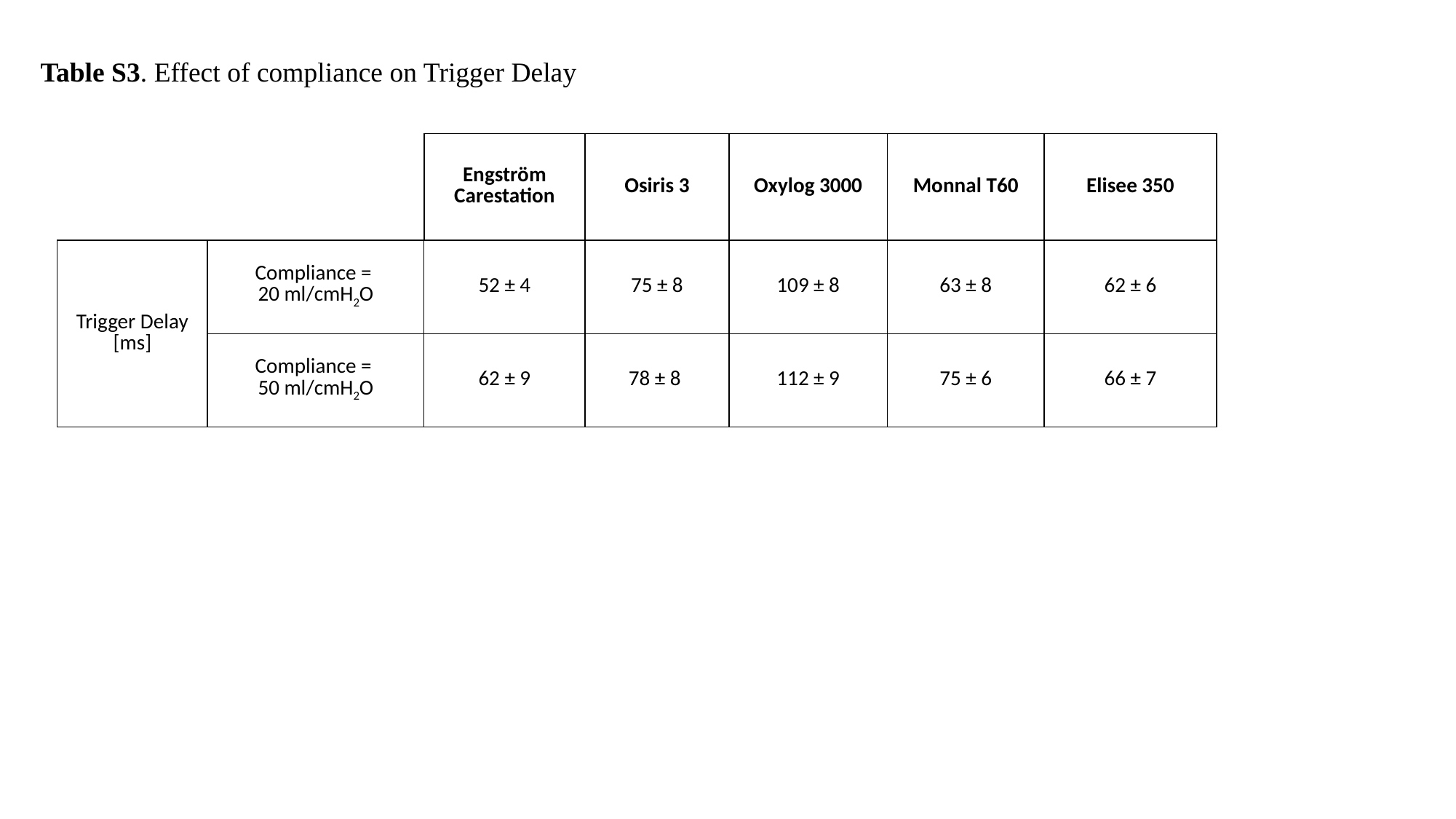

Table S3. Effect of compliance on Trigger Delay
| | | Engström Carestation | Osiris 3 | Oxylog 3000 | Monnal T60 | Elisee 350 |
| --- | --- | --- | --- | --- | --- | --- |
| Trigger Delay [ms] | Compliance = 20 ml/cmH2O | 52 ± 4 | 75 ± 8 | 109 ± 8 | 63 ± 8 | 62 ± 6 |
| | Compliance = 50 ml/cmH2O | 62 ± 9 | 78 ± 8 | 112 ± 9 | 75 ± 6 | 66 ± 7 |

## Slide 5
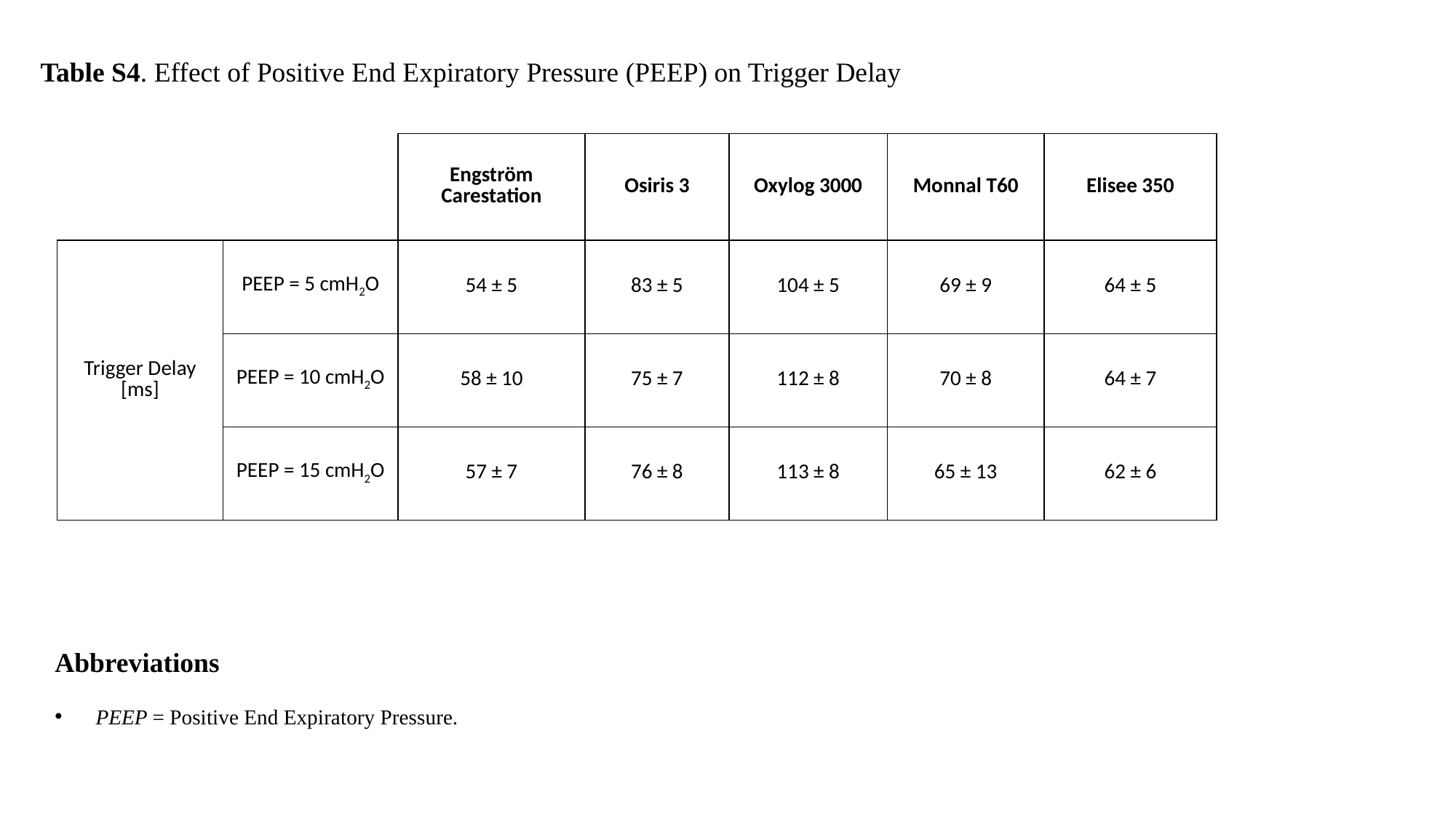

Table S4. Effect of Positive End Expiratory Pressure (PEEP) on Trigger Delay
| | | Engström Carestation | Osiris 3 | Oxylog 3000 | Monnal T60 | Elisee 350 |
| --- | --- | --- | --- | --- | --- | --- |
| Trigger Delay [ms] | PEEP = 5 cmH2O | 54 ± 5 | 83 ± 5 | 104 ± 5 | 69 ± 9 | 64 ± 5 |
| | PEEP = 10 cmH2O | 58 ± 10 | 75 ± 7 | 112 ± 8 | 70 ± 8 | 64 ± 7 |
| | PEEP = 15 cmH2O | 57 ± 7 | 76 ± 8 | 113 ± 8 | 65 ± 13 | 62 ± 6 |
Abbreviations
PEEP = Positive End Expiratory Pressure.

## Slide 6
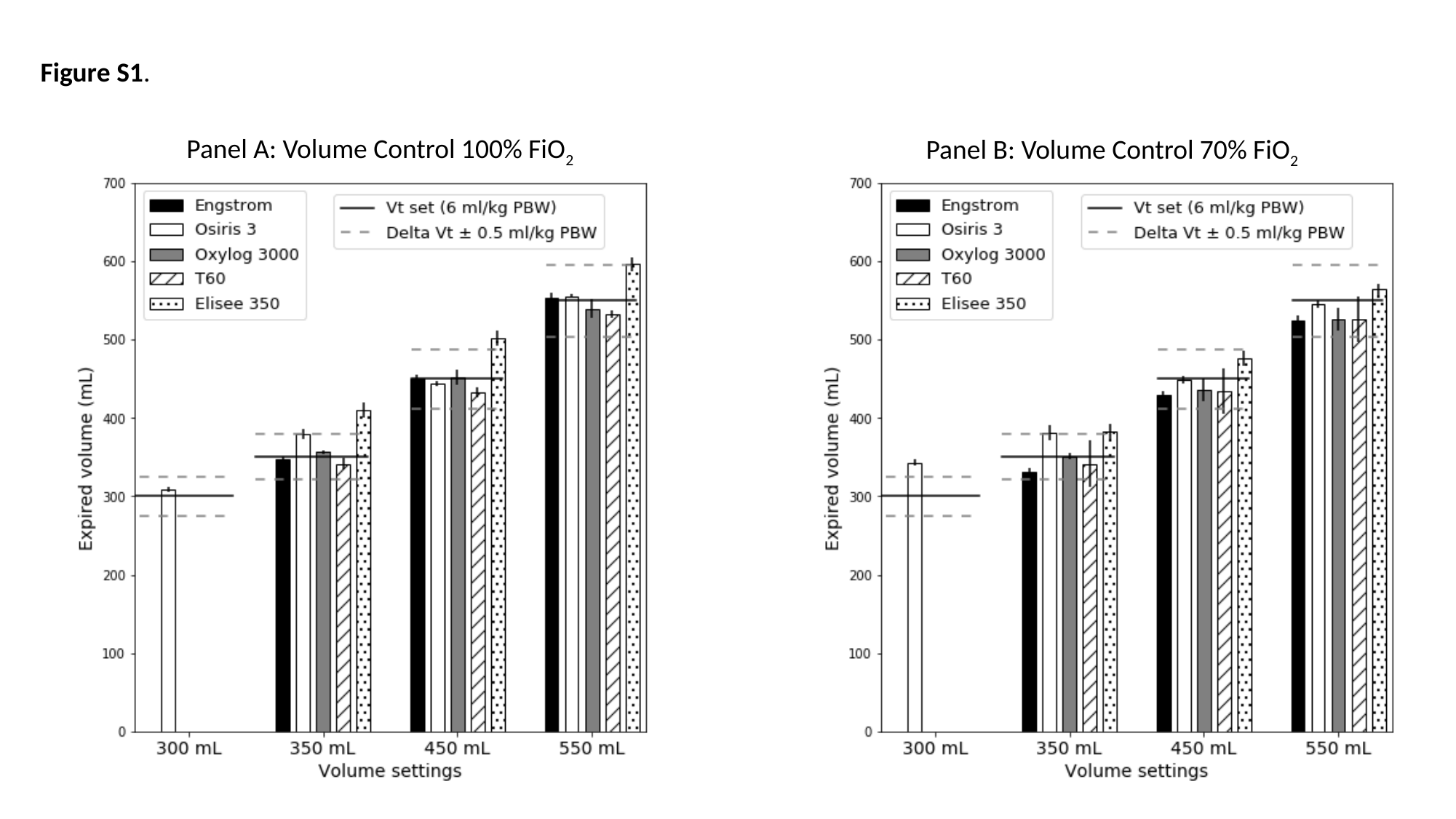

Figure S1.
Panel A: Volume Control 100% FiO2
Panel B: Volume Control 70% FiO2

## Slide 7
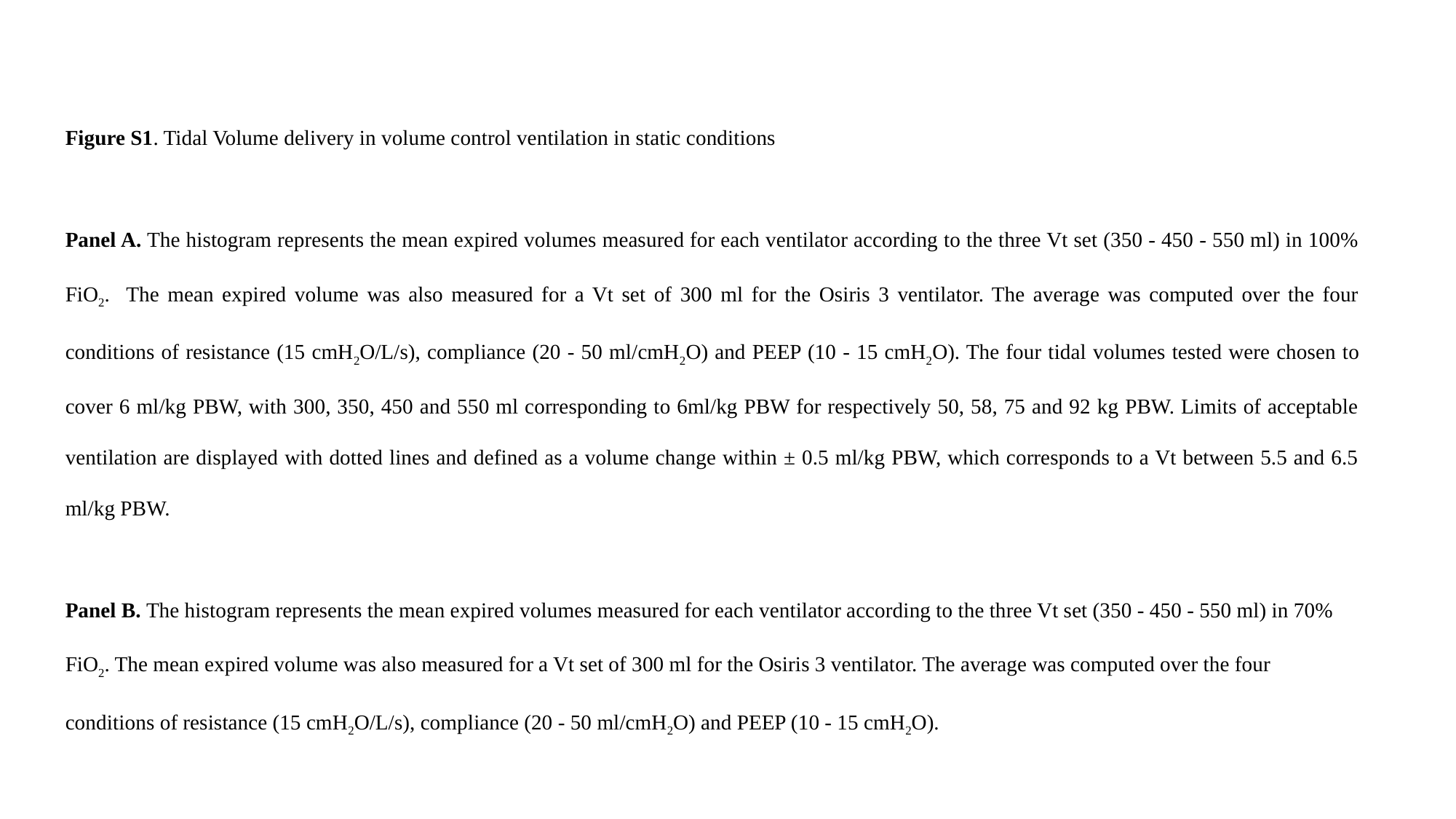

Figure S1. Tidal Volume delivery in volume control ventilation in static conditions
Panel A. The histogram represents the mean expired volumes measured for each ventilator according to the three Vt set (350 - 450 - 550 ml) in 100% FiO2. The mean expired volume was also measured for a Vt set of 300 ml for the Osiris 3 ventilator. The average was computed over the four conditions of resistance (15 cmH2O/L/s), compliance (20 - 50 ml/cmH2O) and PEEP (10 - 15 cmH2O). The four tidal volumes tested were chosen to cover 6 ml/kg PBW, with 300, 350, 450 and 550 ml corresponding to 6ml/kg PBW for respectively 50, 58, 75 and 92 kg PBW. Limits of acceptable ventilation are displayed with dotted lines and defined as a volume change within ± 0.5 ml/kg PBW, which corresponds to a Vt between 5.5 and 6.5 ml/kg PBW.
Panel B. The histogram represents the mean expired volumes measured for each ventilator according to the three Vt set (350 - 450 - 550 ml) in 70% FiO2. The mean expired volume was also measured for a Vt set of 300 ml for the Osiris 3 ventilator. The average was computed over the four conditions of resistance (15 cmH2O/L/s), compliance (20 - 50 ml/cmH2O) and PEEP (10 - 15 cmH2O).

## Slide 8
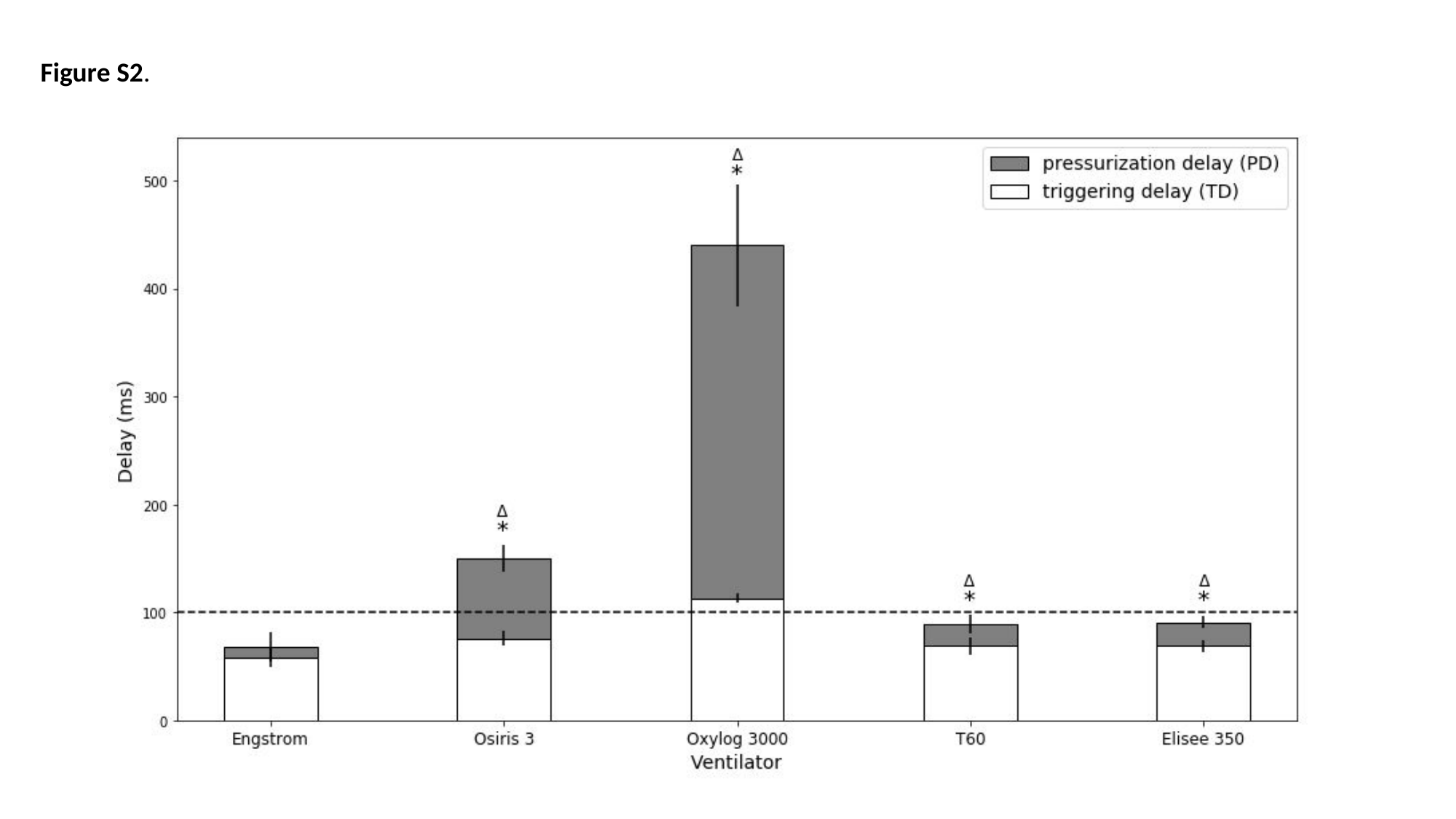

Figure S2.

## Slide 9
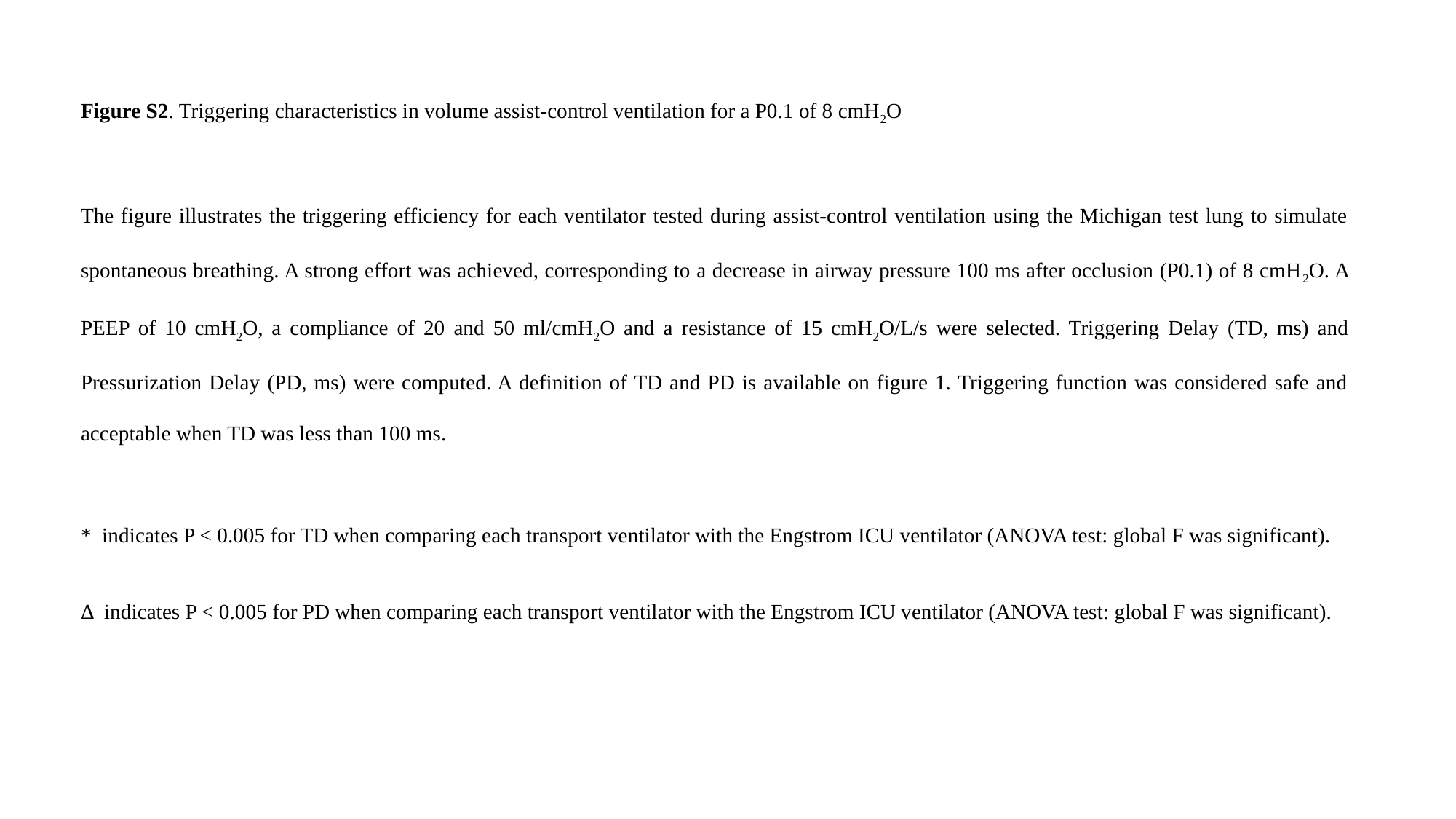

Figure S2. Triggering characteristics in volume assist-control ventilation for a P0.1 of 8 cmH2O
The figure illustrates the triggering efficiency for each ventilator tested during assist-control ventilation using the Michigan test lung to simulate spontaneous breathing. A strong effort was achieved, corresponding to a decrease in airway pressure 100 ms after occlusion (P0.1) of 8 cmH2O. A PEEP of 10 cmH2O, a compliance of 20 and 50 ml/cmH2O and a resistance of 15 cmH2O/L/s were selected. Triggering Delay (TD, ms) and Pressurization Delay (PD, ms) were computed. A definition of TD and PD is available on figure 1. Triggering function was considered safe and acceptable when TD was less than 100 ms.
* indicates P < 0.005 for TD when comparing each transport ventilator with the Engstrom ICU ventilator (ANOVA test: global F was significant).
Δ indicates P < 0.005 for PD when comparing each transport ventilator with the Engstrom ICU ventilator (ANOVA test: global F was significant).
